# Retraction Note: Long noncoding RNA LINC01234 promotes serine hydroxymethyltransferase 2 expression and proliferation by competitively binding miR-642a-5p in colon cancer

**DOI:** 10.1038/s41419-023-05943-5

**Published:** 2023-07-11

**Authors:** Changwei Lin, Yi Zhang, Yifei Chen, Yang Bai, Yi Zhang

**Affiliations:** 1 Department of Gastrointestinal surgery, The Third XiangYa Hospital of Central South University, Changsha, Hunan 410013 China; 2 College of Life Sciences, Central South University, Changsha, Hunan 410078 China; 3 Department of General Surgery, Affiliated Hospital of Xuzhou Medical University, 221000 Xuzhou, P.R. China; 4 Department of Otolaryngology-Head Neck Surgery, The Fourth Hospital of Changsha (The Changsha Affiliated Hospital of Hunan Normal University), Hunan Normal University, Changsha, Hunan 410013 China

Retraction to: *Cell Death and Disease* 10.1038/s41419-019-1352-4, published online 12 February 2019

The Editors-in-Chief have retracted this article because of significant concerns regarding a number of Figures presented in this work, which question the integrity of the data. In Figure 2C there was found to be overlap between the HCT116 sh-NC and Serine images. In Figure 6B there was found to be overlap between the HCT116 NC and Serine images and between the Mimics and Mimics+Serine images. In Figure 8A the image for LOVO miR-642a-5p appears to have been previously published in Figure 7A in the following article [1]. The Editors-in-Chief have therefore lost confidence in the reliability of the results presented in this article. Changwei Lin, Yi Zhang and Yang Bai agree to this retraction. Yifei Chen and Yi Zhang do not agree to this retraction.
